# Whole -genome survival analysis of 144 286 people from the UK Biobank identifies novel loci associated with blood pressure

**DOI:** 10.1097/HJH.0000000000003801

**Published:** 2024-07-10

**Authors:** Sushant Saluja, Rebecca Darlay, Rachel Lennon, Bernard D. Keavney, Heather J. Cordell

**Affiliations:** a Division of Cardiovascular Sciences, Faculty of Biology, Medicine and Health, The University of Manchester; b Division of Medicine and Manchester Academic Health Science Centre, Manchester University NHS Foundation Trust Manchester, Manchester; c Population Health Sciences Institute, Faculty of Medical Sciences, Newcastle University, Newcastle upon Tyne; d Wellcome Centre for Cell-Matrix Research, division of Cell-Matrix biology and regenerative Medicine, School of biological Sciences, Faculty of Biology Medicine and Health, University of Manchester, Manchester Academic Health Science Centre, Manchester, UK

**Keywords:** biobank, blood pressure, genes, hypertension, mendelian randomization, quantitative trait, Saddle Point Approximation Implementation of a Cox Proportional Hazards, survival

## Abstract

This study utilized UK Biobank data from 144 286 participants and employed whole-genome sequencing (WGS) data and time-to-event data over a 12-year follow-up period to identify susceptibility in genetic variants associated with hypertension. Following genotype quality control, 6 319 822 single nucleotide polymorphisms underwent analysis, revealing 31 significant variant-level associations. Among these, 29 were novel – 15 in Fibrillin-2 (*FBN2*) and 4 in Junctophilin-2 (*JPH2*). Mendelian randomization utilizing two identified variants (rs17677724 and rs1014754) suggested that a genetically induced decrease in heart *FBN2* expression and an increase in adrenal gland *JPH2* expression were causally linked to hypertension. Phenome-wide association (PheWAS) analysis using the FinnGen dataset confirmed positive associations of rs17677724 and rs1014754 with hypertension, assessed across 2727 traits in 377 277 individuals. Lastly, rs1014754 positively associated with kallistatin, whereas rs17677724 negatively associated with renin in the Fenland study, suggesting a counterregulatory response to high blood pressure. This study, employing WGS data, identified novel genetic loci and potential therapeutic targets for hypertension.

Hypertension, a leading cause of cardiovascular morbidity and mortality globally, accounted for 17.9 million deaths in 2018. Its incidence doubled from 1990 to 2019, affecting over 1.25 billion individuals [1]. Over 30 genes are implicated in blood pressure (BP) regulation, with rare variants causing monogenic forms of hypertension or hypotension, and more than 1477 common single nucleotide polymorphisms (SNPs) associated with BP [2]. However, current knowledge from genome-wide association studies (GWAS) captures only 27% of the 30–50% estimated heritability of hypertension [2]. This current study addresses these gaps, utilizing UK Biobank's longitudinal data and whole-genome information from 200 005 participants [3]. Unlike traditional GWAS, which often overlook dynamic aspects of hypertension, this study adopts a time-to-event analysis, considering the temporal nature of hypertension onset, potentially revealing predictive genetic variations. The study is distinctive for utilizing whole-genome data and departing from cross-sectional GWAS studies with binary outcomes to identify SNPs associated with hypertension development through a time-to-event analysis (implemented in Saddle Point Approximation Implementation of Cox Proportional Hazards; SPACOX [4]).

The determination of hypertension outcomes in this study incorporated data from various sources within the UK Biobank (UKB), including self-reported data, primary-care records, in-patient hospital data, and first occurrence records (please refer to Supplementary methods).

The UK Biobank contains matched genotypic and phenotypic records for a total of 502 364 participants [3]. At the time of conducting this analysis, whole-genome sequencing data was available for 200 005 participants, constituting the dataset utilized for this study. Stringent quality control measures were implemented to assess sample integrity, including evaluations of missingness, heterozygosity, genotype call rate outliers, sex-discordance, non-European ancestry through principal component analysis, pregnancy status, retracted consent, sex chromosome aneuploidy, and sample relatedness. Additionally, samples with preexisting hypertension before the follow-up period and missing covariate data were excluded from the analysis. Samples passing quality control had their genetic data undergo further stringent genetic quality control measures across 22 autosomal chromosomes. Exclusion criteria included genotype missing rates over 0.1, deviations from Hardy–Weinberg Equilibrium (HWE) at 1 × 10^−15^ significance, variants with minor allele count (MAC) below 100, and minor allele frequency (MAF) under 0.01. Figure 1 provides a summary of this quality control process.

**FIGURE 1:**
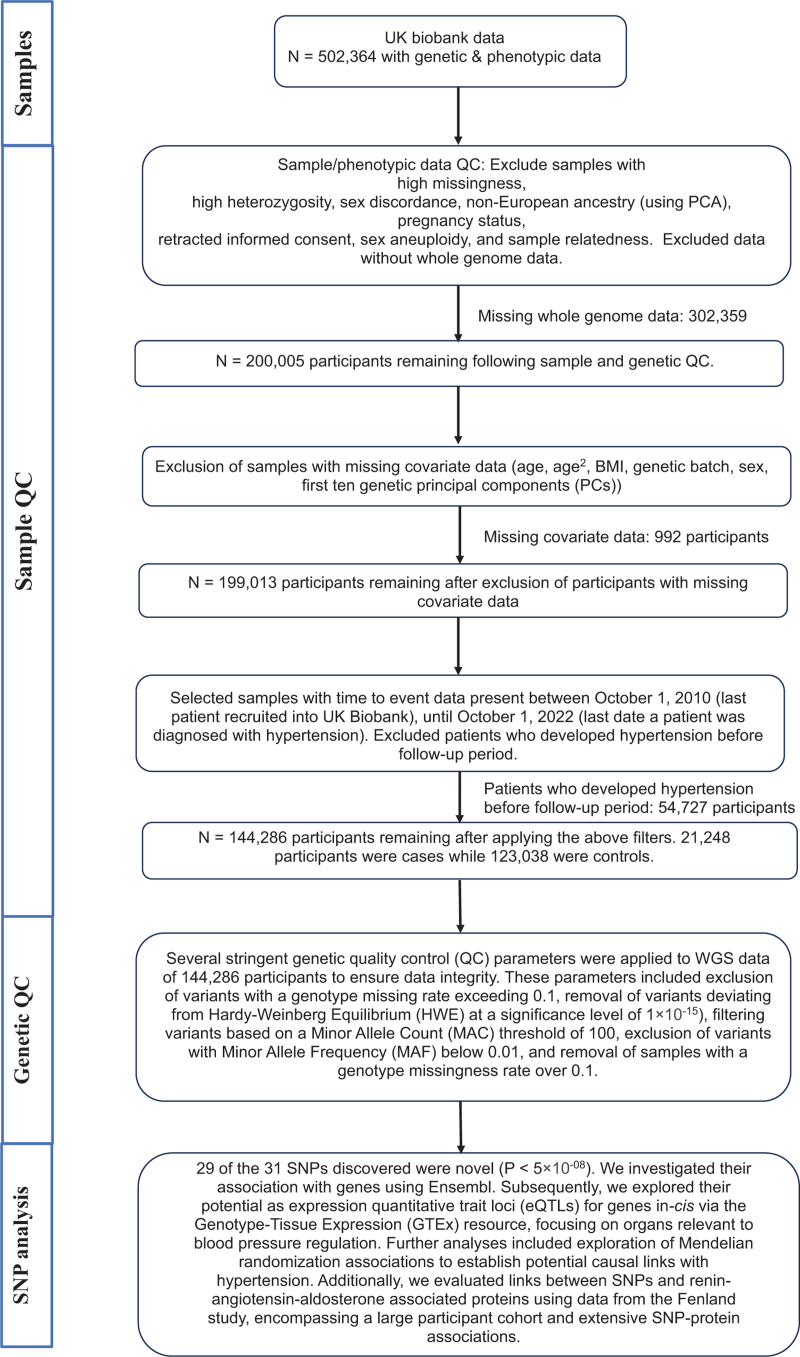
Flowchart representing the sample and genetic quality control process. eQTL, expression quantitative trait loci; GWAS, genome-wide association study; GTEx, genotype-tissue expression; HWE, Hardy–Weinberg equilibrium; MAF, minor allele frequency; MAC, minor allele count; *N*, sample size; PCA, principal component analysis; QC, quality control; UKB, UK Biobank.

The time-to-event analysis commenced from the enrolment of the final UK Biobank participant on 1 October 2010, extending until the latest diagnosis of ‘Essential-hypertension’ within the cohort on 1 October 2022, comprising approximately 12 years (4383 days) of follow-up. The SPACOX package in R-facilitated genome-wide survival analysis, identifying loci associated with hypertension using a saddle point approximation implementation of a Cox proportional hazards regression model [4]. The analysis accounted for the following covariates: age, age^2^, BMI, genetic batch, sex, and 10 principal components, with significant loci identified using a stringent *P* value threshold of less than 5 × 10^−8^.

To discover novel hypertension-associated SNPs, we compared significant SNPs from our study with those previously identified in BP GWAS using the GWAS catalogue (https://www.ebi.ac.uk/gwas/). We utilized Ensembl (https://www.ensembl.org/index.html) to determine the associated genes and investigated whether these SNPs functioned as expression quantitative trait loci (eQTLs) *in-cis*, particularly focusing on organs relevant to BP regulation using Genotype-Tissue Expression (GTEx) resource (https://gtexportal.org/home/) [5]. Further investigation involved interrogating the tissue-dependent Mendelian randomization atlas (http://mrcieu.mrsoftware.org/Tissue_MR_atlas/), to establish potential causal links between identified SNPs and hypertension, spanning 395 complex traits and diseases. Lastly, we evaluated if SNPs causally linked to BP exhibited connections to renin–angiotensin–aldosterone system (RAAS)-associated proteins, utilizing data from the Fenland study, encompassing approximately 10 000 participants, and assessing associations among 10.2 million SNPs and 3892 proteins [6].

After sample quality control, the study included 144 286 UKB participants, with 21 248 hypertension cases and 123 038 controls. Women constituted 83 630 (58%) of the cohort, with an average age of 55.3 ± 0.0279 years, at baseline. The SPACOX analysis identified 31 genome-wide significant SNPs (*P* value <5 × 10^−8^), with the most significant being rs1014754 on chromosome 20, mapping to the Junctophilin-2 (*JPH2*) gene (*P* value = 1.26 × 10^−9^) (Figs. 2 and 3). Twenty-nine of the 31 identified SNP associations were novel, and categorized as follows: 4 (12.9%) as noncoding RNA intronic, 13 (41.9%) as intergenic, and 14 (45.2%) as intronic SNPs. Notably, 20 SNPs were located on chromosome 5, predominantly mapping to the *FBN2* gene (Fig. 3 and Table 1).

**FIGURE 2:**
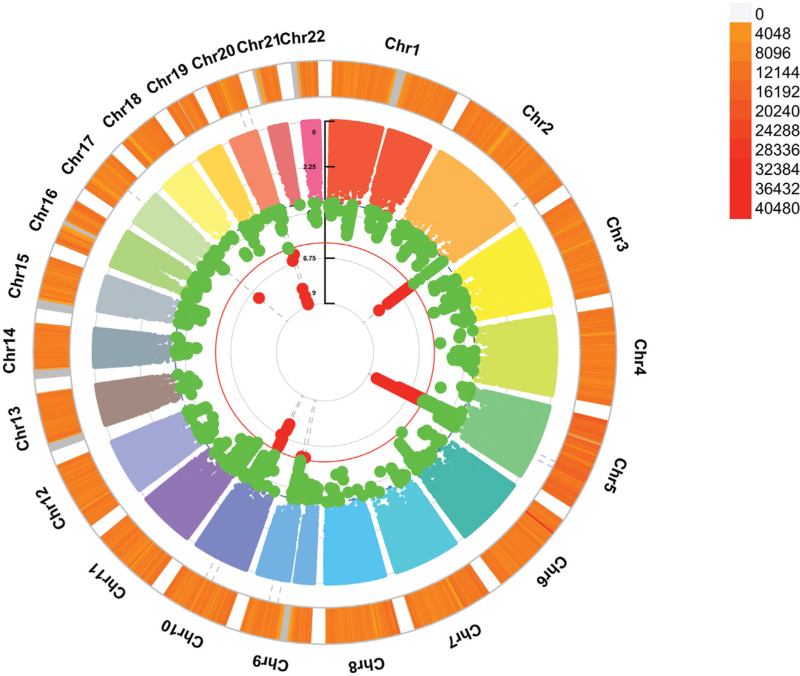
SPACOX whole-genome time-to-event genome-wide association study analysis. The first circos plot at the centre displays the −log_10_*P* values representing the effect of SNP alleles on hypertension. The second circos plot depicts chromosome density. Red dots indicate *P* values less than 5 × 10^−8^. These plots were generated using the ‘CMplot’ R script available at https://github.com/YinLiLin/R-CMplot. GWAS, genome-wide association study; SPACOX, a saddle point approximation implementation based on the Cox PH regression model.

**FIGURE 3:**
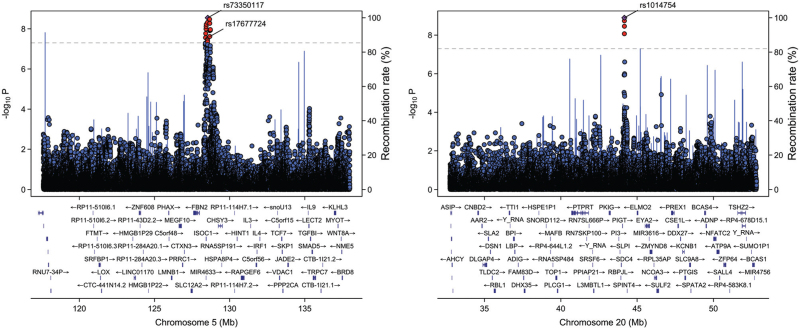
LocusZoom plots display significant associations for genome-wide associations in hypertension. Lead SNPs are denoted by purple diamonds. The left plot illustrates associations around the FBN2 region, with rs17677724 (*P* = 2.41 × 10^−8^) noted as an intergenic SNP and the index, intergenic, SNP rs73350117 (*P* = 2.82 × 10^−9^). On the right, the plot focuses on the *JPH2* region, demonstrating rs1014754 (*P* = 1.26 × 10^−9^), identified as an intronic SNP in the time-to-event GWAS analysis (*N* = 144 286). Linkage disequilibrium (LD) information is depicted as a colour overlay, representing the level of linkage disequilibrium measured by *r*^2^, with red indicating high LD (0.8–1.0) and purple indicating low LD (0.0–0.2). The secondary *y* axis on the right of each plot illustrates recombination rates retrieved from the UCSC (University of California, Santa Cruz) Genome Browser. These plots were generated using the ‘locuszoomr’ R script available at https://github.com/Geeketics/LocusZooms. GWAS, genome-wide association study; LD, linkage disequilibrium.

**TABLE 1:** Significant single nucleotide polymorphisms identified in the time-to-event analysis at a genome-wide association study significance level of 5 × 10^−8^

SNP	Chromosome	Region	POS	Gene	P-(SPACOX)	P-(COX)
rs78881937	2	ncRNA_ intronic	217294055	*DIRC3*	3.85 × 10^−08^	3.78 × 10^−08^
rs398105337	2	ncRNA_ intronic	217308941	*DIRC3*	3.24 × 10^−08^	3.19 × 10^−08^
rs77363583	2	ncRNA_ intronic	217309571	*DIRC3*	9.31 × 10^−09^	9.14 × 10^−09^
rs145903435	2	ncRNA_ intronic	217309640	*DIRC3*	9.51 × 10^−09^	9.34 × 10^−09^
rs4705717	5	Intergenic	115064943	*LOC102467217, CDO1, TRIM36*	4.81 × 10^−08^	4.8 × 10^−08^
rs10060165	5	Intergenic	115065300	*LOC102467217, CDO1, TRIM36*	4.3 × 10^−08^	4.29 × 10^−08^
rs35318989	5	Intergenic	128397037	*FBN2*	2.76 × 10^−08^	2.71 × 10^−08^
rs34490055	5	Intergenic	128408001	*FBN2*	1.71 × 10^−08^	1.68 × 10^−08^
rs36066524	5	Intergenic	128427361	*FBN2*	1.02 × 10^−08^	1 × 10^−08^
rs1561005	5	Intronic	128434669	*FBN2*	7.65 × 10^−09^	7.49 × 10^−09^
rs35581777	5	Intronic	128447854	*FBN2*	5.97 × 10^−09^	5.84 × 10^−09^
rs13177608	5	Intronic	128524837	*FBN2*	3.33 × 10^−08^	3.31 × 10^−08^
rs13177978	5	Intronic	128525091	*FBN2*	3.7 × 10^−08^	3.67 × 10^−08^
rs62390669	5	Intronic	128529377	*FBN2*	4.79 × 10^−09^	4.72 × 10^−09^
rs34952159	5	Intronic	128532401	*FBN2*	4.46 × 10^−08^	4.44 × 10^−08^
rs73350117	5	Intergenic	128542036	*FBN2, SLC27A6*	2.82 × 10^−09^	2.78 × 10^−09^
rs7706547	5	Intergenic	128665200	*FBN2, SLC27A6*	3.35 × 10^−09^	3.3 × 10^−09^
rs111717874	5	Intergenic	128671174	*FBN2, SLC27A6*	5.22 × 10^−09^	5.14 × 10^−09^
rs4836390	5	Intergenic	128675995	*FBN2, SLC27A6*	1.23 × 10^−08^	1.22 × 10^−08^
rs67850503	5	Intronic	128676496	*SLC27A6*	5.35 × 10^−09^	5.26 × 10^−09^
rs35742495	5	Intronic	128679173	*SLC27A6*	1.20 × 10^−08^	1.18 × 10^−08^
rs17677724	5	Intergenic	128679677	*FBN2, SLC27A6*	2.41 × 10^−08^	2.38 × 10^−08^
rs374774125	5	Intronic	128717347	*-*	1.18 × 10^−08^	1.17 × 10^−08^
rs13184883	5	Intergenic	128717853	*FBN2, SLC27A6*	1.09 × 10^−08^	1.08 × 10^−08^
rs76011336	10	Intronic	61789473	*LINC02625*	4.21 × 10^−08^	4.18 × 10^−08^
rs7070797	10	Intergenic	61792015	*LINC02625*	3.62 × 10^−08^	3.57 × 10^−08^
rs72831369	10	Intergenic	61792840	*LINC02625*	4.83 × 10^−08^	4.76 × 10^−08^
rs6031431	20	Intronic	44166512	*JPH2*	8.48 × 10^−09^	8.46 × 10^−09^
rs2179680	20	Intronic	44167210	*JPH2*	1.81 × 10^−09^	1.81 × 10^−09^
rs1014754	20	Intronic	44167334	*JPH2*	1.26 × 10^−09^	1.26 × 10^−09^
rs6031435	20	Intronic	44168718	*JPH2*	3.47 × 10^−09^	3.46 × 10^−09^

It includes the rsIDs, the chromosome on which they are located, their position (in base pairs), the gene to which they map, and the corresponding *P* values for both SPACOX (a Saddle Point Approximation Implementation based on the Cox PH regression model) and Cox analyses. *P*-(SPACOX), *P* value for Cox Proportional Hazards (Saddlepoint Approximation). *P*-(COX), *P*-value for Cox Proportional Hazards (Normal Approximation). *CDO1*, cysteine dioxygenase type 1; *DIRC3*, disrupted in renal carcinoma 3; *FBN2*, fibrillin-2; *JPH2*, junctophilin-2; ncRNA-intronic, noncoding RNA intronic; *P*-(COX), *P* value for Cox Proportional Hazards (normal approximation); POS, position; *P*-(SPACOX), *P*-value for Saddle Point Approximation; SNP, single nucleotide polymorphism; *SLC27A6*, solute carrier family 27 member 6; *TRIM36*, tripartite motif-containing protein 36.

Exploration of the GTEx resource identified 18 eQTLs in the adrenal gland, 16 in the heart-left ventricle and 14 heart-atrial appendage, (Supplementary Figure 1). All SNPs exhibited eQTL effects for *FBN2*, except four that functioned as eQTLs for *JPH2* in the adrenal gland. Mendelian randomization analysis demonstrated a causal association between genetically predicted *FBN2* expression in the heart-left ventricle and hypertension (MR beta = −0.0139011, *P* value = 0.0001660731), as well as a causal link between *JPH2* expression in the adrenal gland and hypertension (MR beta = 0.0141774, *P* value = 3.18939 × 10^−6^). Conditional analysis was carried out using the GCTA-COJO (Genome-wide Complex Trait Analysis – Conditional and Joint Analysis) tool (https://yanglab.westlake.edu.cn/software/gcta/#COJO) in GCTA v1.93 (https://yanglab.westlake.edu.cn/software/gcta/#Download), and identified rs17677724 (mapping to *FBN2*) and rs1014754 (mapping to *JPH2*), as two independent signals.

We performed a phenome-wide association (PheWAS) analysis utilizing the FinnGen consortium (r9.finngen.fi) on the two independent SNPs. This analysis involved 122 996 cases and 289 117 controls for hypertension. Concentrating specifically on SNPs rs17677724 and rs1014754, the effect allele ’T’ linked to reduced *FBN2* expression in the heart-left ventricle (*β* = −0.684, SE = 0.109, *P* value = 3.42 × 10^−9^), exhibited a significant, positive association with hypertension (*β* = 0.492, *P* value = 7.4 × 10^−9^). On the other hand, rs1014754, influencing *JPH2* expression positively in the adrenal gland (*β* = 0.552, *P* value = 3.47 × 10^−8^), similarly demonstrated the effect allele's (‘T’) positive association with hypertension (*β* = 0.0225, *P* value = 4.8 × 10^−5^).

Subsequently, we investigated the relationship between the identified SNPs and the extended RAAS. This system is pivotal in BP regulation and encompasses a range of plasma protein targets [7]. Using a proteogenomic map derived from 10 708 individuals of European descent, encompassing 4775 measured plasma proteins, and identified protein quantitative trait loci (pQTL), we specifically investigated associations with RAAS component proteins [6]. Our findings revealed that rs17677724 exhibited a negative association with renin (*β* = −0.036, SE = 0.018, *P* value = 0.0435). In contrast, rs1014754 displayed a positive association with kallistatin (*β* = 0.031, SE = 0.013, *P* value = 0.0225). These observations suggest a potential counterregulatory mechanism in response to the elevated BP associated with these SNPs.

This study identified novel loci associated with hypertension, including *FBN2* and *JPH2*. Previous studies supporting the correlation of *FBN2* with BP traits, particularly in heart tissue, underscore its role in shaping the passive mechanical properties of major arteries, contributing to early-onset hypertension [8]. *FBN2* demonstrates predominant expression during embryonic and postnatal life. This early-life significance elucidates its association with the early onset of hypertension in our study, explaining its identification by SPACOX [8]. Additionally, our findings highlight the link between increased *JPH2* expression in the adrenal gland and hypertension, a connection that aligns with the pivotal role of tissue-specific voltage-gated L-type calcium channel (LTCC) isoforms in aldosterone biosynthesis [9]. *JPH2* facilitates the recruitment of functional LTCCs to the membrane, plausibly via direct interaction with the channel [9]. Furthermore, it is a prominent blood pressure regulatory locus identified in previous GWAS studies (https://www.ebi.ac.uk/gwas/genes/JPH2), that serves as a crucial structural bridge between the plasma membrane and the sarcoplasmic reticulum, essential for proper excitation–contraction coupling in cardiac muscle cells. Mutations in *JPH2* have been associated with various cardiac conditions, including hypertrophic cardiomyopathy (HCM), dilated cardiomyopathy (DCM), arrhythmias, and sudden cardiac death (SCD) [10]. ClinGen presently designates *JPH2* with moderate evidence supporting its role in HCM under an autosomal dominant inheritance pattern, and likewise for its involvement in other cardiac conditions. Furthermore, moderate evidential backing is observed for its connection with DCM with a semidominant inheritance pattern (https://search.clinicalgenome.org/kb/genes/HGNC:14202).

This study's strength lies in employing a whole genome-wide event time data analysis, using the SPACOX software, to identify significant loci associated with hypertension. In the study conducted by Bi *et al.*[4], imputed data was utilized, whereas we employed WGS data, thereby uncovering novel loci. Variants exclusive to individuals typed solely on SNP arrays could potentially be missed through imputation, leading to unreliable outcomes [11]. Our study presents a significant advancement, yielding more robust results. Additionally, our method's superior ability to detect missed loci in binary phenotyping enhances our genetic insights into hypertension. Validation of variants identified through Mendelian randomization, PheWAS, and examination of their impact on BP-related proteins in the Fenland cohort strengthens our findings. Nevertheless, limitations include the study's focus on middle-aged white participants in the UK Biobank, cautioning against broad generalizations to diverse populations, necessitating validation through diverse study designs [12]. Although we recognize limitations in the SPACOX methodology, such as the inability to account for relatedness through random effects and the determination of effect sizes for SNPs, we note that the methodology is highly scalable and well calibrated for variants across various allele frequencies [4]. Further analyses utilizing alternative approaches such as frailty models incorporating random effects and age-dependent liability threshold (ADuLT) models [13,14] could facilitate comparisons with the SPACOX methodology, validate findings, and offer valuable additional insights. Further investigations are crucial to corroborate our results and determine underlying mechanisms.

## ACKNOWLEDGEMENTS

Author contributions: S.S., R.D., and H.J.C. played significant roles in data acquisition, with S.S. conducting the analysis and drafting the manuscript, under the close supervision of R.D. and H.J.C. H.J.C. supervised the project and led manuscript editing efforts. B.D.K. and R.L. contributed to refining and finalizing the manuscript. All authors provided critical feedback and contributed to shaping the research, analysis, and manuscript.

Data availability statement: the raw genetic and phenotypic data utilized in this study are accessible through the UK Biobank (http://www.ukbiobank.ac.uk/).

This research was funded in whole, or in part, by the Wellcome Trust (Grant numbers 203914/Z/16/Z and 219424/Z/19/Z). For open access, the author has applied a CC BY public copyright licence to any Author Accepted Manuscript version arising from this submission.

Funding statement: this research has been conducted using the UK Biobank Resource under Application Number 46 114 and was funded by the Wellcome Trust and British Heart Foundation (BHF). S.S. is supported by the 4Ward Wellcome Trust Clinical research Training Fellowship (Grant Reference 203914/Z/16/Z). R.L. holds a Wellcome Discovery Award (Grant Reference 227417/Z/23/Z). B.K. holds a BHF Personal Chair (Grant Reference CH/13/2/30154). H.J.C holds a Wellcome Investigator Award in Science (Grant Reference 219424/Z/19/Z).

Ethical approval: the UK Biobank has received ethical approval from the North-West Centre for Research Ethics Committee (Application 11/NW/0382), which applies across the UK. All participants provided informed consent. Further information can be accessed at https://www.ukbiobank.ac.uk/learn-more-about-uk-biobank/about-us/ethics. The research detailed in this manuscript was also approved by UK Biobank (Application 46 114). The data utilized in this article was generated and accessed with approval from the UK Biobank access committee under the application number 46114.

### Conflicts of interest

There are no conflicts of interest.

## Supplementary Material

Supplemental Digital Content

## Supplementary Material

Supplemental Digital Content
