# Supplementary material for: Whole -genome survival analysis of 144 286 people from the UK Biobank identifies novel loci associated with blood pressure
Source: J Hypertens. 2024 Jul 10;42(9):1647–52. doi: 10.1097/HJH.0000000000003801 (PMC11296269; doi:10.1097/HJH.0000000000003801)

Supplementary Figure 1: Heatmap illustrating SNPs functioning as expression-quantitative trait loci (eQTL)-s in *cis* for genes *FBN2* or *JPH2*, denoted within the heatmap tiles. The y-axis represents tissues, while tile colours indicate beta values. Blue signifies negative beta, while red indicates positive beta, with darker shades representing larger absolute values. SNPs are ordered based on the number of organs they function as eQTLs in, while organs are arranged by the total count of e-SNPs (expression SNPs) discovered in our analysis, per tissue.


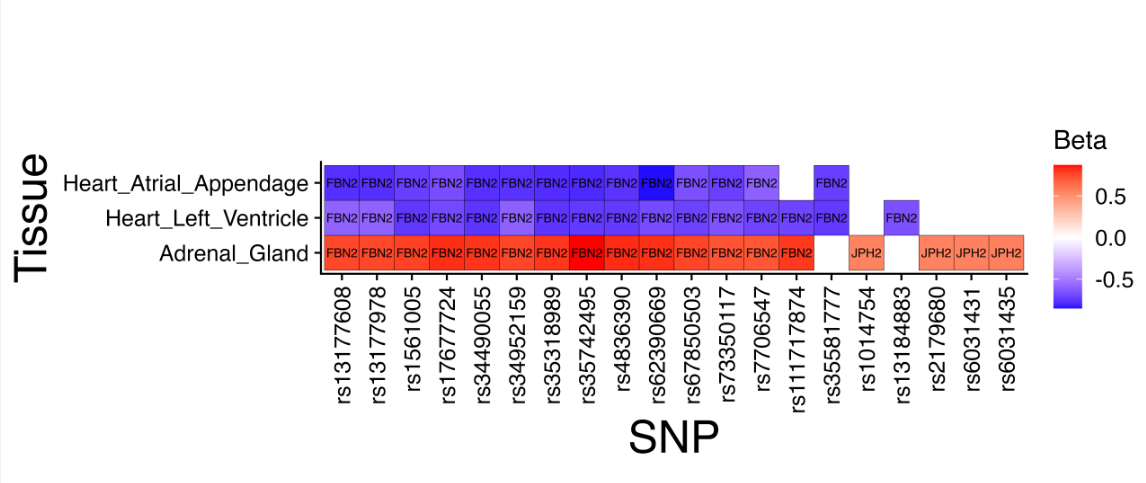

Supplement: Supplemental Digital Content [file jhype-42-1647-s001.doc]
