# Supplementary material for: Whole -genome survival analysis of 144 286 people from the UK Biobank identifies novel loci associated with blood pressure
Source: J Hypertens. 2024 Jul 10;42(9):1647–52. doi: 10.1097/HJH.0000000000003801 (PMC11296269; doi:10.1097/HJH.0000000000003801)
Supplement: Supplemental Digital Content [file jhype-42-1647-s002.docx]

**Supplementary methods**

**Determination of hypertension outcome**

The assessment of hypertension outcomes in this study utilized a variety of data sources from the UK Biobank (UKB). These sources included self-reported information, primary-care records, in-patient hospital data, and records of the first occurrence of hypertension. Participants' hypertension status and medication usage were obtained during the imaging visit through touchscreen questionnaires and nurse interviews. The relevant fields for self-reported diagnosis were Field ID 20002 (Non-cancer illness code, self-reported medical conditions) within Category 100074 (medical conditions self-reported via the touchscreens), which included Codes 1065 (‘hypertension’) and 1072 (‘essential hypertension’). Data collection in Category 100074 was conducted through verbal interviews by trained nurses, covering participants' medical histories, including both past and current conditions such as hypertension and cancer. This dataset also provided details on medical conditions and their respective diagnosis dates. If participants affirmed specific illnesses like angina, asthma, blood clots, chronic bronchitis, diabetes, or high blood pressure, the nurse verified these during the interview. Any discrepancies in the touchscreen questionnaire illness screen (Category ID 1000440) were corrected by the interviewer. Additionally, the interviewer reconfirmed information for participants who reported no major illnesses or disabilities or were uncertain. Medical conditions not assigned a code during the interview were initially recorded as free text and later categorized when possible. We also utilized data from Category ID 100044 (medical-conditions self-reported via touchscreen), which included age at hypertension diagnosis (Field ID 2966) and blood pressure medication use fields (Field IDs 6177 and 6153). Primary care data were extracted from Field ID 42040 (GP Clinical Events record), covering 230,028 participants. This dataset, made available in 2019, includes coded clinical event data from GP system suppliers, such as consultations, diagnoses, procedures, laboratory tests, and prescription data (drug name, quantity, and prescription dates), along with administrative codes for specialist referrals, coded using CTV-3, READ2, and BNF coding systems[1]. Additionally, we used hospital inpatient data available for the entire cohort. This category (2000) includes information on hospital admissions (day cases and overnight stays), diagnoses (including underlying conditions), procedures, and discharge information, available as summary and record-level data, coded using ICD-9, ICD-10, OPCS-3, and OPCS-4 systems[2]. Specifically, diagnosis fields are allocated for each ICD-9 and ICD-10 code, indicating primary and secondary diagnosis positions (Category 2002).

Furthermore, data were retrieved from the UKB death registry, which included the date of death and primary and contributory causes of death, coded using the ICD-10 system. This death-related information is available in Category 100093, titled the Death Summary Report, detailing both the quantity and causes of death within the UK Biobank cohort, identified through linkage with national death registries. Additional fields in this dataset include Age at death (Data-Field 40007), calculated from the date of birth and the date of death; Data-Field 40010, which contains a description of the cause of death; Field 40001, which provides details on the underlying (primary) cause of death using ICD-10; and Field 40002, which outlines contributory (secondary) causes of death using ICD-10. This information is encapsulated within the initial occurrence for health-related outcomes (Category 1712), specifically in the subcategory of circulatory system disorders (Subcategory ID 2409), Field ID 131286 (Date of first reported essential (primary) hypertension). This category includes data on the 'first occurrence' of any code mapped to ICD-10. The data in this field is compiled by mapping read code information from Category 3000 (primary care data), ICD-9 and ICD-10 codes from hospital inpatient data (Category 2000), and ICD-10 codes from the death register (Field 40001, Field 40002), among others. Further details regarding the information contained within these fields and categories can be accessed on the UK Biobank Showcase website at: https://biobank.ndph.ox.ac.uk/showcase/](https://biobank.ndph.ox.ac.uk/showcase/.

**SPACOX Analysis**

For the genome-wide survival analysis, this study utilized the SPACox package in R (https://github.com/WenjianBI/SPACox). This method leverages a saddlepoint approximation based on the Cox proportional hazards (PH) regression model, offering a rapid and precise approach to genome-wide survival analysis[3]. SPACOX combines saddlepoint and normal distribution approximations, selecting the appropriate method based on the proximity of the score statistic to zero. This technique enhances efficiency by fitting a single null Cox PH model for the entire genome, then estimating the empirical Cumulative Generating Function (CGF) of the martingale residuals. The SPA (Saddlepoint Approximation) is then used to adjust the p-values, allowing for the identification of more loci associated with traits such as hypertension compared to traditional genome-wide association studies (GWAS) that use logistic regression[3]. The genome-wide SNP association testing incorporated covariates including age, age squared, Body Mass Index (BMI), sex, and ten principal components representing population structure. Significant SNPs were identified using a rigorous genome-wide significance threshold with a P-value of less than 5×10^-8^. The results, including the Manhattan plot and other relevant figures, were visualized using the "ggplot2" package in R (https://cran.r-project.org/web/packages/ggplot2/index.html).

**Exploration of potential causal mechanisms**

After identifying novel associated SNPs, we used data from Ensembl (https://www.ensembl.org/index.html) to determine the corresponding genes. We then examined whether these SNPs acted as expression quantitative trait loci (eQTLs) for genes in *cis* using the Genotype-Tissue Expression (GTEx) resource (https://www.gtexportal.org/home/). Our investigation focused on organs relevant to blood pressure regulation, such as the kidney, heart, adrenal glands, aorta, and arteries[4,5]. Additionally, we explored the atlas of tissue-dependent Mendelian randomization associations to identify potential causal links between the identified SNPs and hypertension. This web application has established causal connections between gene expression and 395 complex traits, including hypertension [(Non-cancer illness code, self-reported hypertension (UK Biobank)] (http://mrcieu.mrsoftware.org/Tissue_MR_atlas/). The analyses utilized gene expression data from whole blood provided by the eQTLGen (expression Quantitative Trait Loci Genomics Network) consortium (n=31,684), as well as 48 different tissue types from the GTEx project. The methodology is comprehensively detailed in the paper by Richardson et al. (2020)[6]. In essence, their analyses employed the Summary-data-based Mendelian Randomization (SMR) method (v0.710), which operates as a two-sample strategy. Lastly, we investigated whether SNPs causally associated with blood pressure were linked to proteins associated with the RAAS (Renin-Angiotensin-Aldosterone System), which plays a key role in regulating blood pressure and determining target organ damage[7–9]. This analysis used data from the Fenland study, which included around 10,000 participants and examined the association between approximately 10.2 million SNPs (with a minor allele frequency of >1%) and 4775 proteins[10,11].

**Phenome Wide Association Study (PheWAS analysis)**

For the phenome-wide association study (PheWAS), we collected data from the FinnGen database. FinnGen is a public–private partnership research project that integrates imputed genotype data from newly collected and legacy samples from Finnish biobanks, along with digital health record data from Finnish health registries (https://www.finngen.fi/en). The aim of FinnGen is to provide new insights into disease genetics. The FinnGen consortium includes nine Finnish biobanks, various research institutes, universities, university hospitals, 13 international pharmaceutical industry partners, and the Finnish Biobank Cooperative (FINBB) in a pre-competitive partnership[12]. For the PheWAS approach, FinnGen constructed over 2800 endpoints by combining data from various health registers. The endpoints were created using the Finnish versions of the International Classification of Diseases (ICD-8, ICD-9, ICD-10) codes in the Care Register for Health Care and the Causes of Death Register. To enhance the specificity and sensitivity of these endpoints, data from additional registers were incorporated, including the Finnish Cancer Registry (using ICD-O-3 codes), prescription drug purchases (using Anatomical Therapeutic Chemical (ATC) codes), and medication reimbursement data. Additionally, biobanks provided information on smoking status, body mass index (BMI), and gender[12].

The baseline data were collected through various means, either from hospital electronic health records (EHRs) or, in the case of legacy cohorts, from study questionnaires. Clinical expert groups and core FinnGen teams determined the codes used to create each disease endpoint to optimize specificity and sensitivity. For each endpoint, all recorded events with the age at the event were initially documented. Subsequently, first-ever event data were constructed, detailing the age of onset for cases and the age at censoring or the end of follow-up for controls[12]. This data was utilized for the core PheWAS analyses in FinnGen. For our study, we collected phenome-wide association results for variants rs17677724 and rs1014754 from FinnGen release R9 (r9.finngen.fi). This release includes data from 377,277 individuals and 2,272 disease endpoints, which can be explored using the web browser.

References

1 Stroganov O, Fedarovich A, Wong E, Skovpen Y, Pakhomova E, Grishagin I, *et al.* Mapping of UK Biobank clinical codes: Challenges and possible solutions. *PLoS One* 2022; 17:e0275816.

2 Clifton L, Liu X, Collister JA, Littlejohns TJ, Allen N, Hunter DJ. Assessing the importance of primary care diagnoses in the UK Biobank. *European Journal of Epidemiology* 2024; 39:219.

3 Bi W, Fritsche LG, Mukherjee B, Kim S, Lee S. A Fast and Accurate Method for Genome-Wide Time-to-Event Data Analysis and Its Application to UK Biobank. *Am J Hum Genet* 2020; 107:222–233.

4 Guyton AC, Coleman TG, Cowley AV, Scheel KW, Manning RD, Norman RA. Arterial pressure regulation. Overriding dominance of the kidneys in long-term regulation and in hypertension. *Am J Med* 1972; 52:584–594.

5 Mensah GA, Croft JB, Giles WH. The heart, kidney, and brain as target organs in hypertension. *Cardiol Clin* 2002; 20:225–247.

6 Richardson TG, Hemani G, Gaunt TR, Relton CL, Davey Smith G. A transcriptome-wide Mendelian randomization study to uncover tissue-dependent regulatory mechanisms across the human phenome. *Nat Commun* 2020; 11:185.

7 Te Riet L, van Esch JHM, Roks AJM, van den Meiracker AH, Danser AHJ. Hypertension: renin-angiotensin-aldosterone system alterations. *Circ Res* 2015; 116:960–975.

8 Arendse LB, Danser AHJ, Poglitsch M, Touyz RM, Burnett JC, Llorens-Cortes C, *et al.* Novel Therapeutic Approaches Targeting the Renin-Angiotensin System and Associated Peptides in Hypertension and Heart Failure. *Pharmacol Rev* 2019; 71:539–570.

9 Danser AH, van Kats JP, Admiraal PJ, Derkx FH, Lamers JM, Verdouw PD, *et al.* Cardiac renin and angiotensins. Uptake from plasma versus in situ synthesis. *Hypertension* 1994; 24:37–48.

10 Lindsay T, Westgate K, Wijndaele K, Hollidge S, Kerrison N, Forouhi N, *et al.* Descriptive epidemiology of physical activity energy expenditure in UK adults (The Fenland study). *Int J Behav Nutr Phys Act* 2019; 16:126.

11 Pietzner M, Wheeler E, Carrasco-Zanini J, Cortes A, Koprulu M, Wörheide MA, *et al.* Mapping the proteo-genomic convergence of human diseases. *Science* 2021; 374:eabj1541.

12 Kurki MI, Karjalainen J, Palta P, Sipilä TP, Kristiansson K, Donner KM, *et al.* FinnGen provides genetic insights from a well-phenotyped isolated population. *Nature* 2023; 613:508–518.
